# 6-Gingerol alleviates placental injury in preeclampsia by inhibiting oxidative stress via BNIP3/LC3 signaling-mediated trophoblast mitophagy

**DOI:** 10.3389/fphar.2023.1243734

**Published:** 2023-10-13

**Authors:** Anna Li, Man Zhao, Zexin Yang, Zhenya Fang, Weiyi Qi, Changqing Zhang, Meijuan Zhou, Junjun Guo, Shuxian Li, Xietong Wang, Meihua Zhang

**Affiliations:** ^1^ Key Laboratory of Birth Regulation and Control Technology of National Health Commission of China, Shandong Provincial Maternal and Child Health Care Hospital Affiliated to Qingdao University, Jinan, China; ^2^ Department of Clinical Medicine, Shandong First Medical University, Jinan, China; ^3^ Department of Obstetrics and Gynecology, Provincial Hospital Affiliated to Shandong First Medical University, Jinan, China

**Keywords:** 6-gingerol, preeclampsia, mitophagy, trophoblast, oxidative stress

## Abstract

**Background and aims:** Preeclampsia (PE) is the leading cause of maternal and fetal morbidity and mortality worldwide. Apoptosis of trophoblast cells induced by oxidative stress is a principal reason of placental injury in PE. 6-Gingerol, an antioxidant from ginger, plays an important role in many disease models, but its effect on obstetric diseases has not been elucidated. In this study, we investigated the protective effect of 6-gingerol against placental injury.

**Methods:** *In vitro* hypoxia/reoxygenation (H/R) model of HTR8/Svneo cells and preeclamptic mice model were established to simulate PE. The effects of 6-Gingerol on PE were evaluated by morphological detection, biochemical analysis, and Western blot.

**Results:** We found that H/R treatment induced cell apoptosis, increased the production of reactive oxygen species, malondialdehyde and lactate dehydrogenase, and decreased superoxide dismutase in trophoblast. In addition, the polarization of mitochondrial membrane potential and the cellular calcium flux were also destroyed under H/R condition, which also activated BCL2-interacting protein 3 (BNIP3) and provoked excessive mitophagy. Importantly, 6-Gingerol reversed these corrosive effects. Furthermore, the placenta damage in PE-like mouse caused by the cell apoptosis, oxidative stress and mitophagy was mitigated by 6-Gingerol.

**Conclusion:** These findings suggest that 6-Gingerol exerts a protective effect against placental injury in PE by reducing oxidative stress and inhibiting excessive mitophagy caused by mitochondrial dysfunction.

## 1 Introduction

Preeclampsia (PE), as a common pregnancy complication, is the leading cause of maternal and fetal morbidity and mortality, affecting up to 8% of pregnancies worldwide (Ives et al., 2020). The placenta is the direct link between the mother and fetus, which plays a crucial role in exchanging oxygen and nutrients. Therefore, normal placental development is the guarantee of fetal growth and development. During the implantation period of the embryo, extravillous trophoblasts invade the maternal decidua, and engraft and remodel the spiral, which is critical to proper placental development (Abbas et al., 2020). Inadequate placental function caused by the obstruction of this process results in pregnancy complications, such as PE, fetal growth restriction (FGR), and recurrent spontaneous abortion (RSA). Placental malperfusion is the main manifestation of PE, which disrupts the balance between reactive oxygen species (ROS) and antioxidants, known as oxidative stress, due to hypoxia. Several papers found that oxidative stress in PE was predictable and could be developed as a therapeutic target for PE clinically (Liu D. et al., 2021; Colson et al., 2021; Guerby et al., 2021).

Mitochondria, involved in redox homeostasis, Ca^2+^ homeostasis, apoptosis, and various other cellular processes, are exquisitely sensitive to hypoxia and oxidative stress. It has been shown that ischemia and excessive ROS caused by improper spiral artery remodeling were closely related to mitochondrial dysfunction in PE development (Zussman et al., 2020; Giussani, 2021; Yang et al., 2021). Mitophagy is defined as the selective degradation of damaged or dysfunctional mitochondria by autophagy. Defects of mitophagy could result in the accumulation of cellular ROS and eventually lead to the development of preeclampsia (Ausman et al., 2018).

BNIP3 (BCL2 and adenovirus E1B 19-kDa-interacting protein 3) is involved in the receptor-dependent mitophagy pathway, serving as an inducible receptor in response to hypoxia and other stimuli (Tang et al., 2019; Vara-Pérez et al., 2021). BNIP3 could directly bind to LC3 to induce both cell death and autophagy (Xu et al., 2019). Similar to autophagy, LC3II transformed from LC3I located on the membrane of autophagosomes mediated by p62 is the primary signaling in mitophagy, which regulated the digestion of damaged mitochondria by autophagosomes. A previous study revealed that BNIP3 dysregulation was associated with impaired placental mitophagy and oxidative stress during the development of PE (Zhou et al., 2021).

6-Gingerol is a major compound extracted from ginger, which exhibits anti-inflammatory, antioxidant, anticancer, and antiapoptotic properties (Zhang et al., 2018; Hong et al., 2020; Han et al., 2022). In a variety of ischemia-reperfusion diseases, 6-gingerol could significantly reduce proinflammatory factors, and inhibit apoptosis and oxidative stress in various tissues, such as in the intestine, cerebrum, and myocardium (Zhang et al., 2016; Ma et al., 2021; Zhao et al., 2021). In addition, 6-gingerol was found to effectively improve atherosclerosis and lung injury (Hong et al., 2021; Hu et al., 2023). However, the protective effects of 6-gingerol against placental dysfunction and its associated obstetric complications, such as PE, FGR, and RSA, are still unclear. Therefore, we investigated the rescuing effect of 6-gingerol on oxidative stress injury of the placenta and its mechanisms through improving the disorder of mitophagy caused by oxidative stress.

## 2 Material and methods

### 2.1 Human subjects

All participants were recruited from the Maternal and Child Health Care Hospital of Shandong Province, affiliated to Qingdao University, all of whom had signed informed consents. Ethical approval was obtained from the Ethics Committee of Maternal and Child Health Care Hospital of Shandong Province, affiliated to Qingdao University. A total of 10 women with early-onset PE and 10 age-matched control subjects, following cesarean section, were enrolled in the study. The diagnostic criteria of PE were as follows (Cohen and Friedman, 2015): normal blood pressure before pregnancy and the first experience of hypertension (systolic blood pressure ≥160 mmHg or a diastolic blood pressure ≥110 mmHg on at least 2 occasions) and proteinuria (≥2 g/24 h or 3 + by dipstick in two random samples collected at > 4-h intervals) after 20 weeks of gestation. According to the onset time of clinical signs, early-onset PE was defined as earlier than the 34th week (Raymond and Peterson, 2011). The exclusion criteria of participants were as follows: hypertensive disorders; neurological, endocrinological, or other systemic diseases; intrauterine fetal death; fetal genetic defects; or pregnancy with the assistance of reproductive technologies. The placental tissue was subjected to flash freezing in liquid nitrogen for protein extraction or paraformaldehyde fixation. The clinical information is shown in Table 1.

**TABLE 1:** Clinical characteristics of the pregnant women enrolled in this study.

Patient characteristic	Normal pregnancy (n = 10)	Preeclampsia (n = 10)	*p*-value
Maternal age (years)	29.8 ± 3.88	29.0 ± 3.50	0.4332
Gestational age (weeks)	39.57 ± 0.86	33.98 ± 2.0	<0.0001
Body mass index (kg/m^2^)	28.0 ± 4.93	29.6 ± 3.92	0.3467
Systolic blood pressure (mm/Hg)	114.7 ± 6.6	161.5 ± 2.59	<0.0001
Diastolic blood pressure (mm/Hg)	69.8 ± 8.12	77.2 ± 10.28	<0.0001
Neonatal birth weight (g)	3555 ± 381.4	1959 ± 704.5	<0.0001
Urine protein (g/24 h)	-	+	
Smoking status	No	No	
Abnormal fetus	No	No	

Data are presented as mean ± S.E.M, and the significant difference between groups was analyzed by Student’s t-test.

### 2.2 Preeclamptic mouse model preparation

The preeclamptic mouse model was established following a modified protocol, as described previously (Jing et al., 2018). Briefly, 10-week-old C57BL/6 mice, female and male species, purchased from the Laboratory Animal Center of Shandong University, were maintained on a 12 h/12 h dark and light cycle at 18°C–22°C with free access to food and water.

Female mice were mated with male mice in a 2:1 ratio, and plug discovery was considered to be gestation day GD 0.5. Pregnant mice were randomly divided into three groups: CT (n = 6), L-NAME (n = 6), and L-NAME + 6-gingerol (n = 6). The mice in the L-NAME and L-NAME + 6-gingerol groups received subcutaneous injections of 125 mg/kg/day L-NAME (MedChemExpress, USA) from GD 9.5 to GD 18.5. The L-NAME + 6-gingerol group was also intraperitoneally administered with 2 mg/kg 6-gingerol (MedChemExpress, USA) from GD 8.5 to GD 18.5, as shown in Figure 6A (Zhang et al., 2018; Zhao et al., 2021). The CT group was injected with the same amount/volume of saline intraperitoneally and subcutaneously during the same period. Maternal systolic blood pressure (SBP) and diastolic blood pressure (DBP) were monitored by tail-cuff plethysmography using the BP-2010A Blood Pressure Analysis System (Softron, Beijing, China) on GD 14.5 and GD 17.5. Urine samples were collected on GD 17.5 for 24 h of pregnancy and analyzed for protein content by BCA assay (23227, Thermo Fisher, USA). The relevant data are presented in Supplementary Figure S2. On GD 18.5, mice were sacrificed by cervical dislocation, and placental weights, number of fetuses, and fetal weights were recorded. Placentae were collected and stored at −80°C or fixed with 4% paraformaldehyde for the next analysis. All animal experiments were approved by the Ethical Committee of Maternal and Child Health Care Hospital of Shandong Province, affiliated to Qingdao University.

### 2.3 Cell culture and treatment

The human trophoblasts cell line HTR8/SVneo (HTR8) was purchased from the American Type Culture Collection. The cells were cultured in Roswell Park Memorial Institute 1640 (RPMI 1640) medium (Invitrogen, California, USA) supplemented with 10% fetal bovine serum (GIBCO, New Zealand) and 1% penicillin–streptomycin (Solarbio, China). The hypoxia/reoxygenation (H/R) *in vitro* model was established by placing the cells in a hypoxia condition (4% N_2_/5% CO_2_/1% O_2_) for 12 h and then subjecting them to reoxygenation (5% CO2/95% air) for 12 h, prior to pretreatment with 6-gingerol, MitoTEMPO, or transfection.

HTR8 cells were co-transfected with Mitochondrial-RFP and EGFP-LC3 plasmid to measure the mitophagy flux, following the instructions of the manufacturer (GENE, China). Cell images of EGFP-LC3 and Mito-RFP were captured with the ImageXpress^®^ Micro Confocal System (Molecular Devices, USA) at 24 h after transfection.

Then, 50 nM MitoTracker Green (Beyotime, C1046), 50 nM LysoTracker Red (Beyotime, C1046), and 5 μg/mL Hoechst (Beyotime, C1027) were used to label the mitochondria, lysosome, and nucleus, respectively, according to the manufacturer’s instructions. The images were captured using the ImageXpress^®^ Micro Confocal System (Molecular Devices, USA).

### 2.4 Immunohistochemistry

The placental tissues from human and mouse models were fixed in a 4% paraformaldehyde solution for subsequent paraffin embedding. After dewaxing and rehydrating using graded ethanol, antigen retrieval pretreated using water-bath heating was applied to the sections. Then, non-specific antigen blocking was performed with goat serum (10%) for 30 min at room temperature. Subsequently, the sections were incubated in primary antibodies (1:200) overnight at 4°C. After washing with PBS, the placental tissues were incubated with secondary antibodies for 1 h at 37°C and developed with diaminobenzidine tetrahydrochloride, and the signal was measured using an inverted fluorescence microscope (Olympus, BX53F, Japan).

### 2.5 Cell viability

The Cell Counting Kit-8 (CCK-8) (CK04-100 T, Solarbio, China) was used to assess cell viability, according to the manufacturer’s recommendations. A measure of 10 μL of CCK-8 solution per well was added to cells seeded in a 96-well plate for 1 h at 37°C, and then, absorbance at 450 nm was detected using a microplate reader. Each experiment was repeated six times.

### 2.6 EdU assay

The proliferation of HTR8 cells was detected with EdU staining, as described previously. Then, 50 μL of the EdU (10 µM) reagent (C0075, Beyotime, China) was added to cells seeded in the 96-well plate for 2 h at 37°C to label the cells. The cells were fixed in 4% paraformaldehyde solution for 15 min and then permeabilized with 0.3% Triton X-100 for 15 min. After incubation with the click-reaction reagent for 30 min at room temperature in the dark, Hoechst 33342 was added to counterstain the nucleus. The cell images were captured and analyzed by the ImageXpress^®^ Micro Confocal System (Molecular Devices, USA).

### 2.7 Detection of cell apoptosis

An Annexin V-fluorescein isothiocyanate (APC)/propidium iodide (PI) apoptosis detection kit (A6012, Uelandy, China) was used to determine the ratio of apoptotic cells by flow cytometry, according to the manufacturer’s instructions. Briefly, HTR8 cells with the indicated treatment were collected and incubated with Annexin V-FITC and PI staining solution. Apoptosis was then analyzed using a flow cytometer with standard optics (FACS Caliber; Becton Dickinson, Heidelberg, Germany).

### 2.8 TUNEL staining

According to the manufacturer’s instructions, TUNEL staining with an *in situ* cell death detection kit (Roche, Basel, Switzerland) was used to examine the break of nuclear DNA as an index of apoptosis in paraffin-embedded sections of placental tissues from human and mouse models. Images were captured and quantified using the ImageXpress^®^ Micro Confocal System with five random fields from each section.

### 2.9 ROS and mtROS detection

2′,7′-Dichlorofluorescin diacetate (DCFH-DA, Beyotime, China) was used to measure total intracellular ROS levels. Approximately 7 × 10^3^ cells were seeded in a 96-well plate. After H/R and other pretreatment processes, the cells were detached by trypsinization or directly incubated with 100 μL of DCFH-DA (10 μM) dissolved in RPMI 1640 medium without FBS for 30 min at 37°C in a dark environment. After washing with serum-free medium three times, the cells were analyzed by flow cytometry (FACS Caliber; Becton Dickinson, Heidelberg, Germany) to detect the mean fluorescence intensity (MFI) or observed using the ImageXpress^®^ Micro Confocal System (Molecular Devices, USA). Accordingly, the ROS levels of each group were quantified and compared.

To detect mitochondrial ROS production, HTR8 cells seeded in 6-well plates at a density of 2×10^5^ per well were treated, as described previously. MitoSOX™ Red Mitochondrial Superoxide Indicator was used, as described previously. Briefly, MitoSOX™ reagent (5 μM) was supplied to cells for 10 min at 37°C. After washing three times gently, the fluorescence intensity of the MitoSOX™ reagent in the cells was visualized using the ImageXpress^®^ Micro Confocal System (Molecular Devices, USA).

### 2.10 Lipid peroxidation (MDA) assay

Lipid peroxidation levels were detected in 10 mg mouse placental tissue or 2 × 10^6^ HTR8 cells with a malondialdehyde (MDA) test kit (S0131, Beyotime, China), according to the manufacturer’s instructions. Mouse placenta tissue or HTR8 cells were homogenized with lysis buffer, and the supernatant was obtained by centrifugation at 10,000 g for 10 min. The protein content in tissues or cells was measured with a BCA protein quantitative kit. The MDA detection working solution was added to the supernatant. After boiling for 15 min, the supernatant was collected by centrifugation at 1000 g at room temperature for 10 min. The absorbance at 532 nm was measured using a microplate reader (Synergy H1, BioTek, USA). The unit protein concentration MDA content was counted and calculated.

### 2.11 LDH release assay

LDH leaking into the culture medium was detected, according to the manufacturer’s instructions of the LDH Release Assay Kit (C0016, Beyotime, China). Briefly, the HTR8 cells seeded onto a 96-well plate were treated with 6-gingerol or MitoTEMPO for different periods and then cultured in H/R conditions. After the indicated treatment, the cell culture supernatant was collected and centrifuged at 400 g for 5 min to discard cell debris. Then, 120 μL of the supernatant was transferred to a clean 96-well plate with an addition of 60 μL reaction mixture, followed by incubation for 30 min at room temperature in the dark. The absorbance at 490 nm was measured using a microplate reader (Synergy H1, BioTek, USA).

### 2.12 Oxidative stress assessment

After the indicated treatment, HTR8 cells and culture medium supernatants were collected. Levels of superoxide dismutase (SOD) and total glutathione (GSH) were detected using a detection kit (Beyotime, Beijing, China), according to the manufacturer’s instructions. The relative levels were detected and analyzed on the microplate reader (Synergy H1, BioTek, USA).

### 2.13 Mitochondrial transmembrane potential (MMP) measurement

The determination of mitochondrial membrane potential by JC-1 staining was carried out, according to the manufacturer’s instructions. After the aforementioned treatment, HTR8 cells seeded in 6-well plates were collected and incubated with the 1×JC-1 working staining solution at 37°C for 30 min. After washing with the detection buffer two times, the cells were re-suspended in 150 µL of the RPMI 1640 medium. Red and green fluorescence were visualized and quantized using the ImageXpress^®^ Micro Confocal System (Molecular Devices, USA).

### 2.14 Transmission electron microscopy

Transmission electron microscopy (TEM) was used to examine the ultrastructural features of HTR8 cells with the indicated treatment. The cells were fixed with 2.5% glutaraldehyde and collected for transportation and then postfixed with 1% OsO_4_ for 2 h, followed by dehydration using graded ethanol. After infiltration and embedding in epoxy resin, cells were cut into slices of 60–80 nm, stained with uranyl acetate and lead citrate, and then subjected to TEM for capturing images.

### 2.15 Western blot analysis

Cells and tissues were lysed in RIPA buffer (Solarbio, Beijing, China) containing a proteinase inhibitor cocktail (Beyotime, Beijing, China) and quantified using the BCA kit (Solarbio, Beijing, China). Equal proteins were separated in SDS-PAGE and electrophoretically transferred onto polyvinylidene fluoride (PVDF) membranes. After immersing in 5% non-fat milk for 1 h at room temperature, the membranes were incubated with primary antibodies with a dilution of 1:1000 overnight at 4°C. The membranes rinsed with TBST were hybridized with the horseradish peroxidase-labeled secondary antibody for 1 h at room temperature the next day. An enhanced chemiluminescence detection kit (Amersham LifeScience, Buckinghamshire, United Kingdom) was used to display the protein bands. The expression of target proteins was quantified by normalizing to *β*-actin. Independent experiments were performed at least three times, and the representative pictures were presented.

Cleaved caspase-3 antibody (9664), BCL-2 (124), and LC3A/B (12741) were purchased from Cell Signaling Technology (USA). TOMM20 (ab56783) antibodies were purchased from Abcam (USA). *β*-Actin (6600901) was purchased from Proteintech (China). Horseradish peroxidase-labeled goat-anti-mouse immunoglobulin G (GB23301) and horseradish peroxidase-labeled goat-anti-rabbit immunoglobulin G (GB23303) were purchased from Servicebio (China).

### 2.16 Statistical analysis

Data are presented as the mean ± standard error of least three different biological replicates. Student’s t-test was performed to compare two groups of data. Multiple comparisons (≥3) were performed using one-way analysis of variance (ANOVA), followed by the Tukey–Kramer multiple comparison test. The quantified results were visualized using bar charts. *p* < 0.05 was considered significant.

## 3 Results

### 3.1 BNIP3 activation and apoptosis in trophoblasts are associated with PE

**FIGURE 1:**
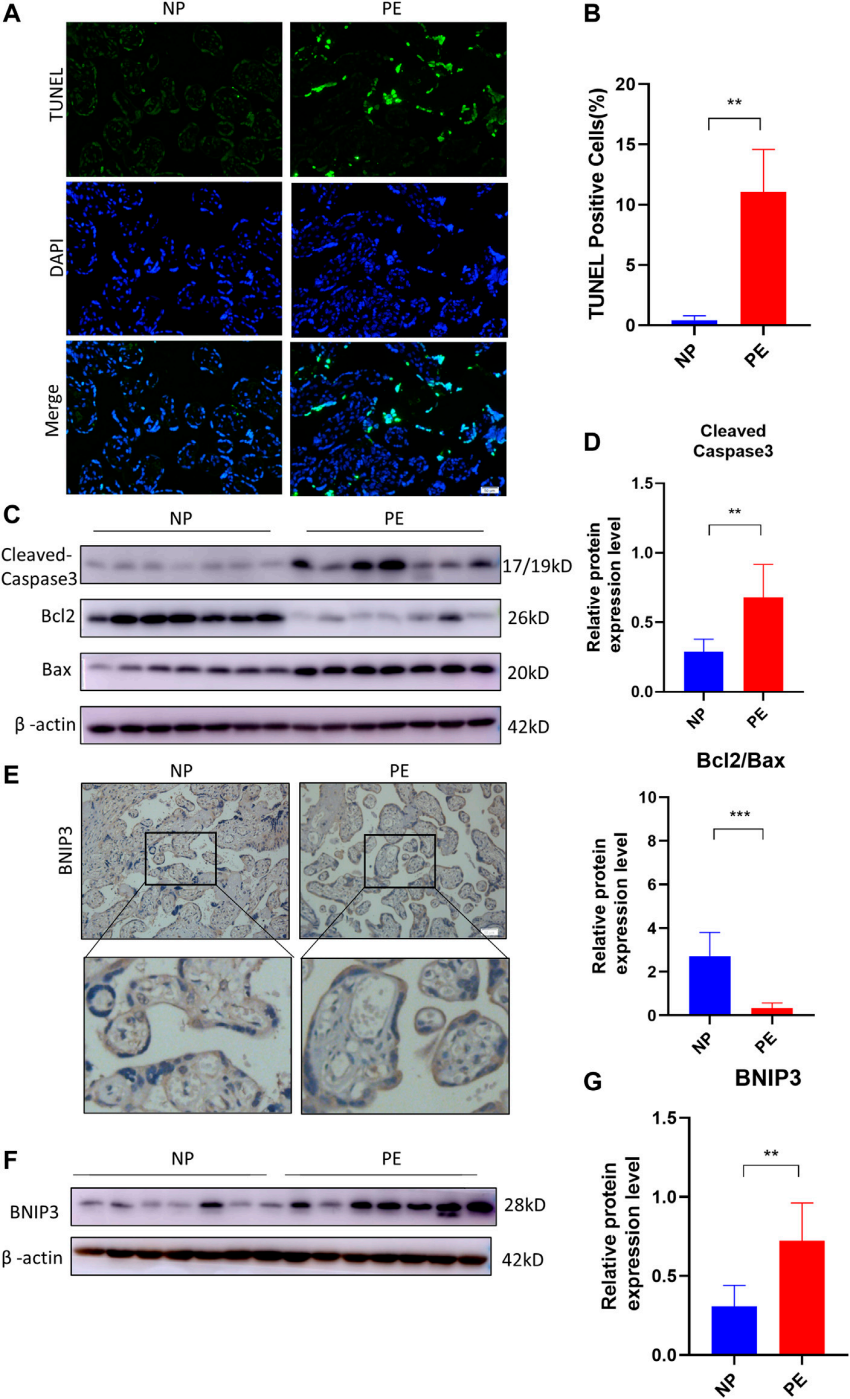
Association of BNIP3, placental apoptosis, and PE. Placentas of normal (NP) (n = 10) and PE (n = 10) pregnant women were collected and analyzed. **(A, B)** Representative placental images and corresponding semiquantification of TUNEL assay. Scale bar, 50 μm. **(C, D)** Western blots and corresponding semiquantification of Cleaved Caspase-3, Bcl2, and Bax in NP and PE placentae. **(E)** Representative images of BNIP3 of NP and PE placentae detected by immunohistochemical staining. Scale bar, 50 μm. **(F, G)** Western blots and corresponding semiquantification of BNIP3 in NP and PE placentae. The data are shown as mean ± SD and analyzed by Student’s t-test based on at least three independent experiments. ns, no significance; *p < 0.05, **p < 0.01, ***p < 0.001, and ****p < 0.0001.

The human placentae were collected from normal pregnant (NP) (n = 10) and PE (n = 10) women, according to clinical diagnostic criteria. TUNEL assay was used to detect placental apoptosis. As shown in Figures 1A, B, TUNEL-positive cells in PE placentae were more than those in NP placentae. Subsequently, we detected the expression of apoptosis-related proteins in human placentae. There were significant increases in the levels of Bax, Cleaved Caspase-3 and decrease in the level of Bcl2 in PE placentae compared to NP placentae (Figures 1C, D). Next, we analyzed the expression of mitophagy protein in PE placenta. The result of immunochemistry staining in Figure 1E showed the expression of BNIP3 increased in the trophoblast of the placenta of the PE group compared to that of the NP group. Consistent with previous data, the protein level of BNIP3 increased significantly in PE placenta tissue compared to that of NP (Figures 1F, G). These results demonstrated that increased apoptosis of placental trophoblast cells is accompanied by activation of mitophagy mediated by BNIP3 in PE placentae.

### 3.2 6-Gingerol alleviates H/R injury by inhibiting apoptosis

**FIGURE 2:**
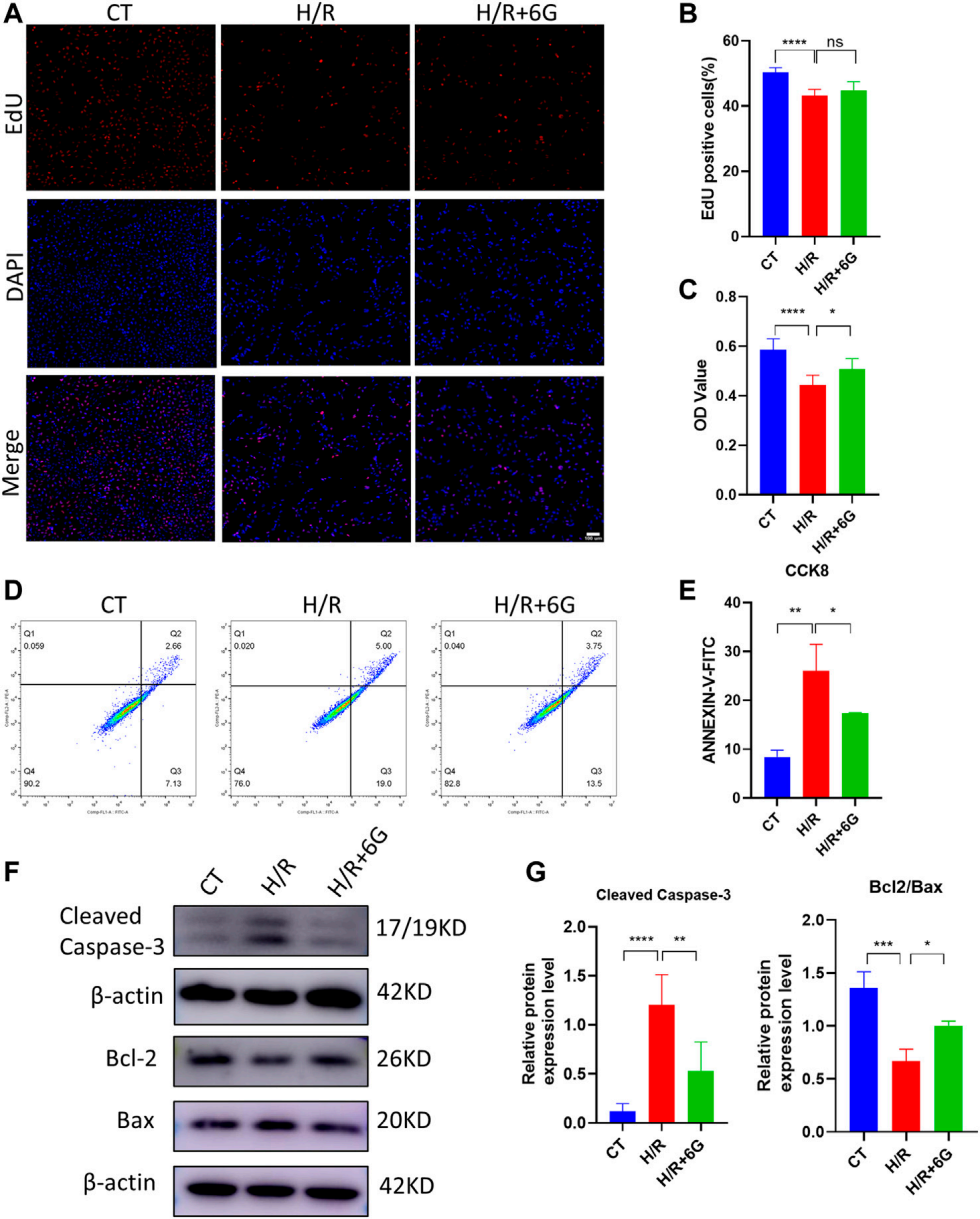
6-Gingerol inhibited the apoptosis of HTR8 cells induced by H/R treatment. HTR8 cells were pretreated with 6-gingerol for 4 h and exposed to the indicated hypoxia condition. The proliferation and viability were detected using EdU **(A, B)** and CCK8 **(C)** assays. Scale bar, 100 μm. **(D, E)** The staining of Annexin V-FITC and PI was performed to determine cell apoptosis using a flow cytometry assay (n = 3). Typical results **(F)** and statistical results **(G)** of Western blotting for Cleaved Caspase-3, Bcl2, and Bax in HTR8 cells. The data are shown as mean ± SD and analyzed by a one-way ANOVA test, followed by the Tukey–Kramer multiple comparison test based on at least three independent experiments. ns, no significance; *p < 0.05, **p < 0.01, ***p < 0.001, and ****p < 0.0001.

Subsequently, HTR8 cells were treated *in vitro* with H/R to mimic the hypoxic damage in preeclampsia. To investigate the role of 6-gingerol in PE, the HTR8 cells were treated with 6-gingerol before subjection to H/R treatment. 6-Gingerol was found to have no effects on the viability of HTR8 cells in concentrations up to 20 μM (Supplementary Figure S1B). The CCK-8 test was performed to check the effect of 6-gingerol on H/R injured cell proliferation. 6-Gingerol showed a protective effect on HTR8 under the H/R condition. Based on the result in Supplementary Figure S1C, we chose a concentration of 10 μM in the following examinations. The results showed that cell proliferation in the H/R treated group exhibited a significant decrease compared to the control group, whereas 6-gingerol administration dramatically improved viability of HTR8 cells compared with the H/R treated group (Figures 2A–C). Annexin V-FITC/PI double-staining revealed that H/R increased the apoptosis rate of HTR8 cells, which was reduced by 6-gingerol (Figures 2D, E). In support of this, WB results showed that the expression of apoptosis markers, such as Cleaved Caspase-3, BCL2, and BAX, was markedly increased by the H/R treatment but could be reversed by 6-gingerol (Figures 2F, G).

### 3.3 6-Gingerol protects against oxidative injury induced by H/R

**FIGURE 3:**
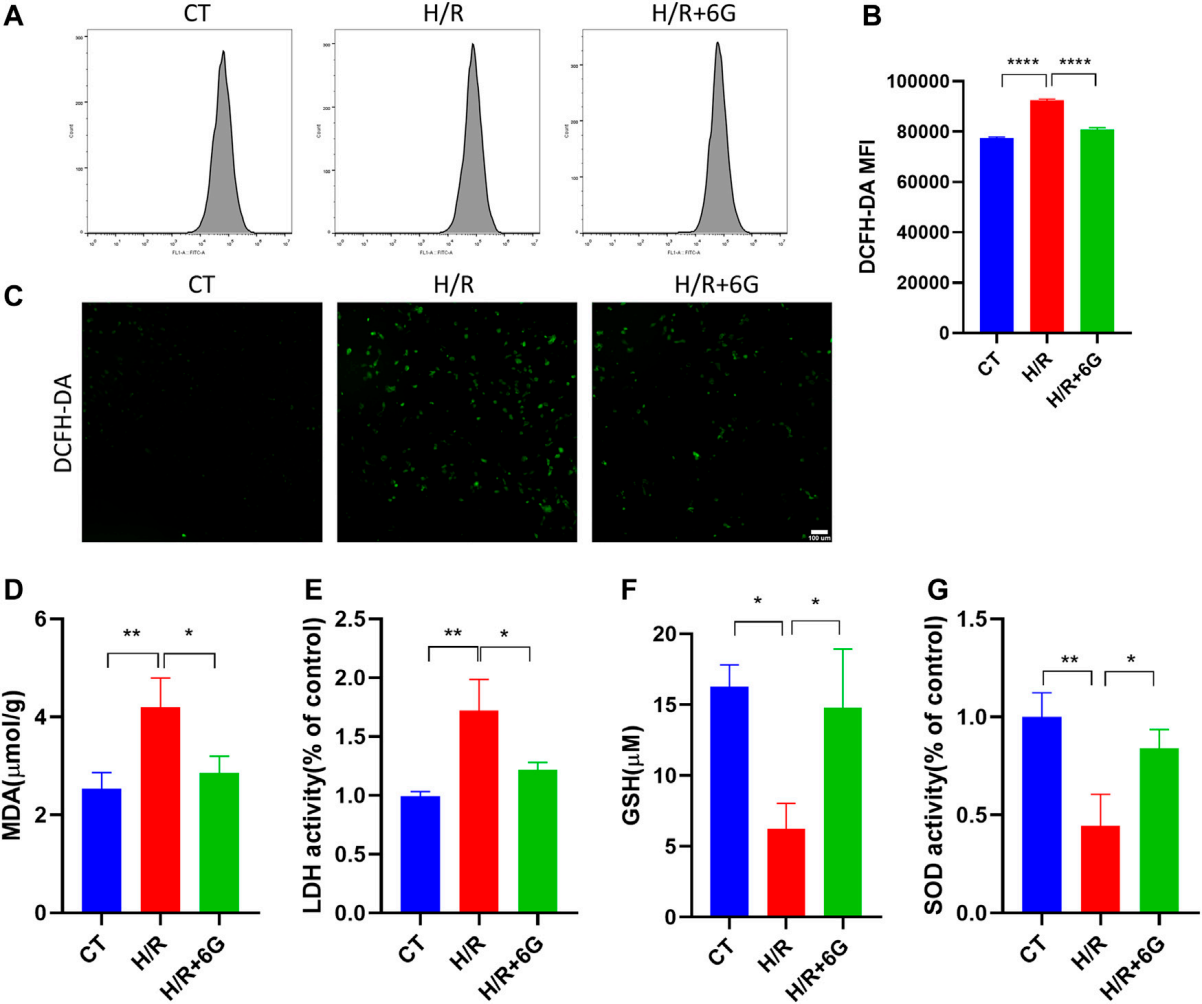
Effect of 6-gingerol on H/R-generated oxidative stress injury in HTR8 cells. DCFH-DA was used to determine intracellular ROS levels in HTR8 cells treated with or without 6-gingerol under the H/R condition by flow cytometry **(A, B)** and fluorescence microscopy **(C)**. The histogram shows the mean ROS production. Scale bar, 100 mm. **(D)** MDA level, **(E)** LDH release, **(F)** GSH level, and **(G)** SOD activity. The data are shown as mean ± SD and analyzed by a one-way ANOVA test, followed by the Tukey–Kramer multiple comparison test based on at least three independent experiments. *p < 0.05, **p < 0.01, and ****p < 0.0001.

To investigate the effect of 6-gingerol on the redox system, we examined the biomarkers of oxidative stress, such as ROS, MDA, LDH, GSH, and SOD. The cytoplasmic superoxide anion level was detected using DCFH-DA fluorescent probes, which showed that 6-gingerol affects ROS production in trophoblasts under the H/R condition (Figures 3A–C). MDA, a sign of mitochondrial respiratory chain destruction, and leakage of LDH, paralleled that of ROS (Figures 3D, E). On the other hand, as antioxidative molecules, the GSH and SOD levels decreased in the H/R-treated HTR8 cells compared to the control (CT) group, which was reversed by 6-gingerol (Figures 3F, G). The GSH level revealed that 6-gingerol rescued the reduction of glutathione caused by H/R. These data indicated that a large amount of ROS accumulated in HTR8 cells caused by H/R, and 6-gingerol relieved the oxidative injury.

### 3.4 6-Gingerol reduces H/R-initiated mitochondrial damage

**FIGURE 4:**
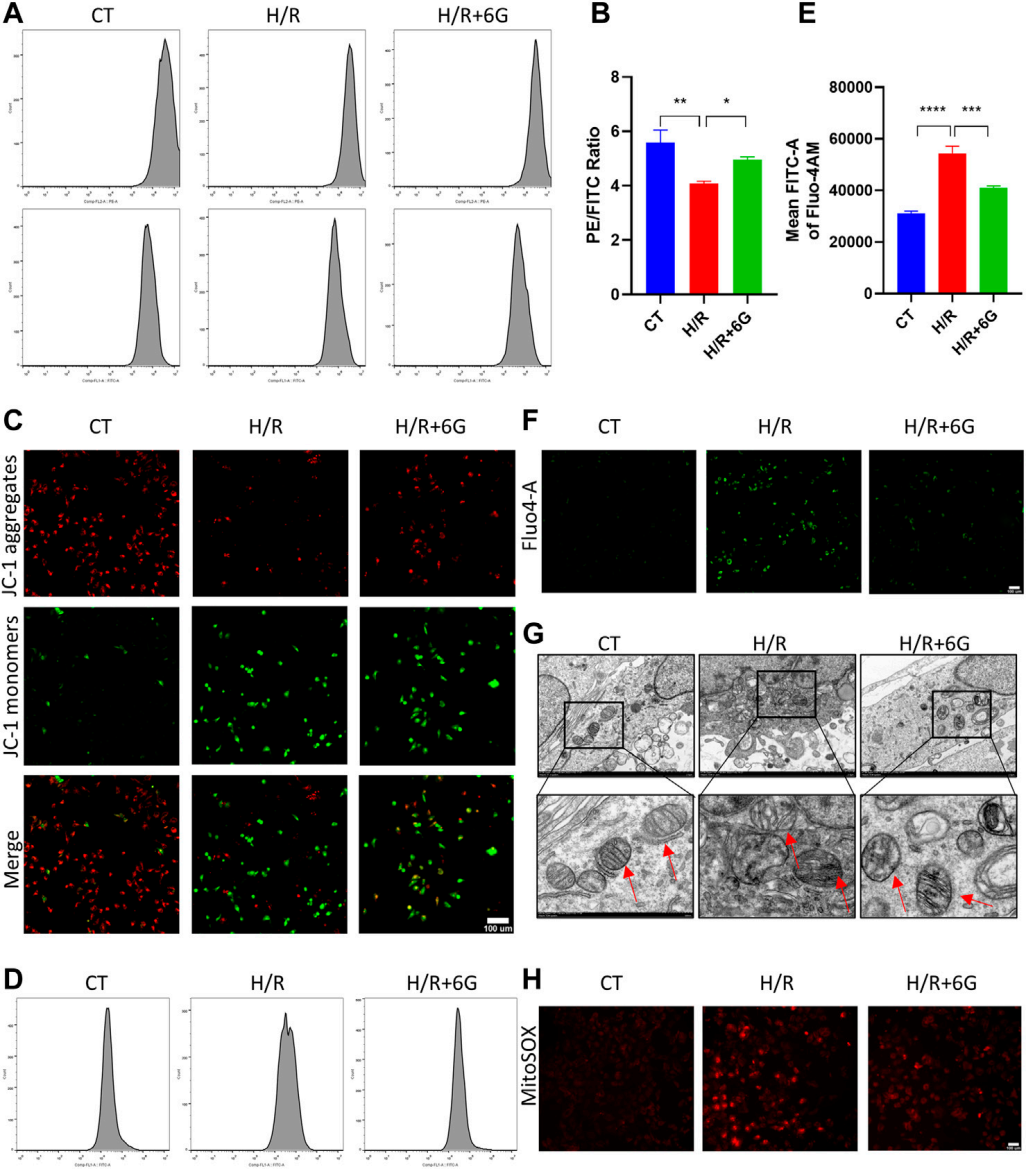
6-Gingerol attenuated the damage of mitochondria. MMP was evaluated by JC-1 staining, using flow cytometry **(A, B)** and photograph **(C)**. Scale bar, 100 µm. The intracellular free Ca^2+^ level of HTR8 cells was determined by cytometry **(D, E)** and monitored using a fluorescence microscope **(F)**. Scale bar, 100 µm. **(G)** Representative transmission electron microscopy (TEM) images of HTR8 cells. The red arrow represents mitochondria. Scale bar, 100 µm. **(H)** Representative images of MitoSOX Red staining for detection of mitochondrial superoxide. Scale bar, 100 µm. The data are shown as mean ± SD and analyzed by a one-way ANOVA test, followed by the Tukey–Kramer multiple comparison test based on at least three independent experiments. *p < 0.05, **p < 0.01, ***p < 0.001, and ****p < 0.0001.

To further explain the effects of 6-gingerol on HR-initiated oxidative injury, we focused on mitochondrial function. To explore the disruption of mitochondria due to H/R, MMP was detected by JC-1 staining. As shown in Figures 4A–C, MMP significantly decreased after H/R treatment, which implied the mitochondrial was depolarized. In addition, the depolarization of MMP was attenuated by 6-gingerol. As abnormal mitochondrial function can cause calcium transportation imbalance, the intracellular free Ca^2+^ was examined by Fluo-4 AM. As shown in Figures 4D–F, the intracellular free Ca^2+^ level of the H/R group was lower compared to that of the CT group. 6-gingerol plays a vital role in rescuing calcium flow imbalance. Moreover, TEM scanning was used to detect ultrastructural mitochondrial changes (Figure 4G). The results showed that the number of mitochondria decreased, the mitochondria swelled, and the rupturing of mitochondrial cristae after H/R treatment. Administration of 6-gingerol mitigated the aforementioned variations and improved the ultrastructure of mitochondria. Subsequently, to investigate whether H/R-induced ROS were derived from mitochondria, we used MitoSOX Red to measure the level of mtROS because mitochondrial ROS (mtROS) was the main source of intracellular ROS. MitoSOX Red fluorescence was dramatically increased in the HTR8 cells after H/R injury compared with the CT group, indicating H/R-induced ROS formation in mitochondria. As shown in Figure 4H, 6-gingerol significantly inhibited mtROS formation, as evidenced by reduction of the red fluorescence intensity. All the findings indicate that 6-gingerol reduces structural and functional damage of mitochondria in trophoblasts induced by H/R treatment.

### 3.5 6-Gingerol inhibited excessive mitophagy to defend mitochondrial function from H/R-induced injury

**FIGURE 5:**
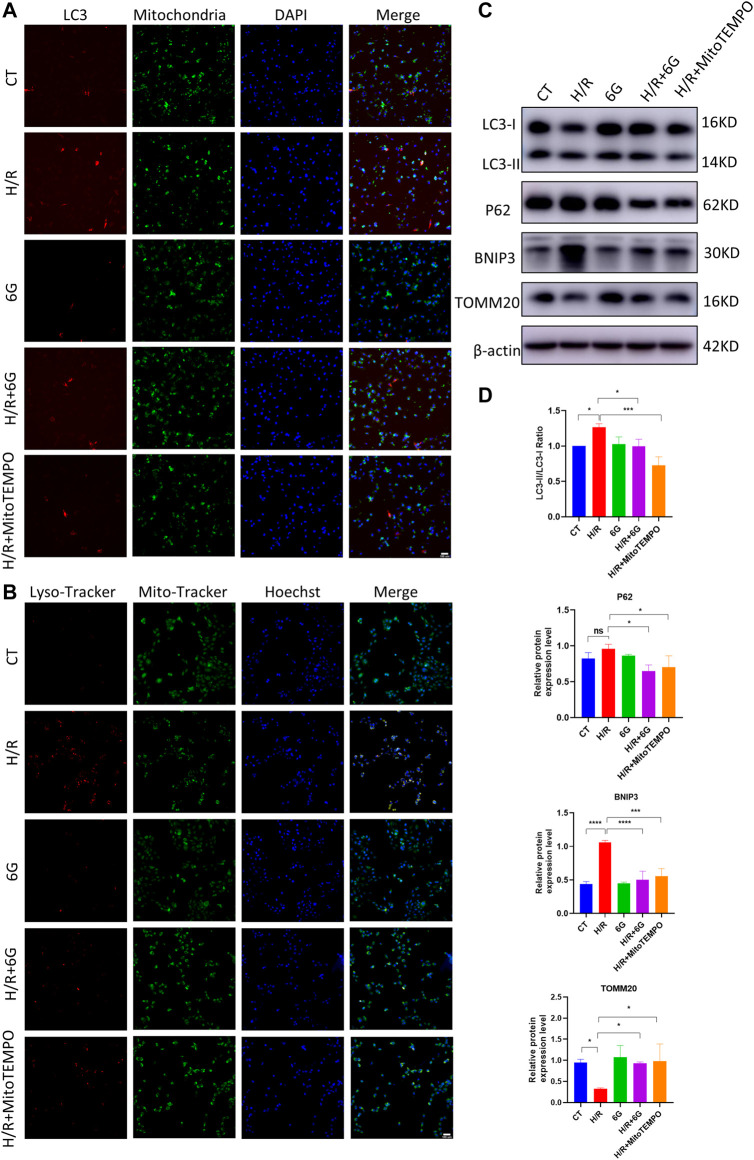
6-Gingerol protected HTR8 cells against excessive mitophagy. HTR8 cells co-transfected with Mito-RFP and EGFP-LC3 plasmids were pretreated with 6-gingerol or mitoTEMPO for 4 h and exposed to the indicated hypoxia condition. **(A)** Representative images. Scale bar, 100 µm. HTR8 cells were pretreated with 6-gingerol or mitoTEMPO for 4 h and exposed to the indicated hypoxia condition. **(B)** Representative staining of MitoTracker and LysoTracker. Scale bar, 100 µm. **(C, D)** Western blots and corresponding semiquantification of LC3, P62, BNIP3, and TOMM20. The data are shown as mean ± SD and analyzed by a one-way ANOVA test, followed by the Tukey–Kramer multiple comparison test based on at least three independent experiments. *p < 0.05, **p < 0.01, ***p < 0.001, and ****p < 0.0001.

A previous study demonstrated mitochondrial damage induced mitophagy. To further explain the effects of 6-gingerol on H/R injury, we focused on mitophagy, which drives cellular death via excessive self-consumption. In order to confirm that the effects of 6-gingerol on H/R injury are due to its antioxidant activity, we further detected the effects of MitoTEMPO, a mitochondrial ROS scavenger, on mitophagy under the H/R condition. To assess the formation of mitophagosomes, HTR8 cells were co-transfected with Mito-RFP and EGFP-LC3 plasmid for tracking potential colocalization between mitochondria and autophagosomes. H/R treatment markedly increased colocalization between mitochondria and LC3-labeled autophagosomes, whereas 6-gingerol decreased the colocalization (Figure 5A).

To investigate the fusion of mitophagosomes with lysosomes, which is the final step of mitophagy, MitoTracker and LysoTracker probes were used to label mitochondria and lysosomes, respectively. As shown in Figure 5B, the colocalization of MitoTracker and LysoTracker as an overlap of red and green fluorescence increased dramatically under the H/R condition, which was diminished by pretreatment with 6-gingerol. Furthermore, the results of HTR8 cells indicated that H/R treatment downregulated the mitochondria marker TOMM20 and upregulated the expression of BNIP3 and the LC3II/LC3I ratio (Figures 5C, D). However, the expression of BNIP3 and the LC3II/LC3I ratio was reduced by 6-gingerol. As a mitochondrial ROS inhibitor, MitoTEMPO was used to further identify the effects of mitochondrial ROS on mitophagy under the H/R condition. The WB results showed that MitoTEMPO blocked the initiation of mitophagy by reducing the expression of BNIP3 and LC3. Interestingly, P62 did not decrease in the H/R group, which accumulated due to the impaired fusion of autophagosomes–lysosomes, demonstrating H/R-impaired autophagy flux. 6-Gingerol, as well as MitoTEMPO, restored p62 to degradation. Together, these results indicated that 6-gingerol moderates H/R-induced mitophagy by inhibiting the production of mitochondrial ROS.

### 3.6 6-Gingerol reduced placenta dysfunction, mitochondrial damage, and apoptosis in PE-like mice

**FIGURE 6:**
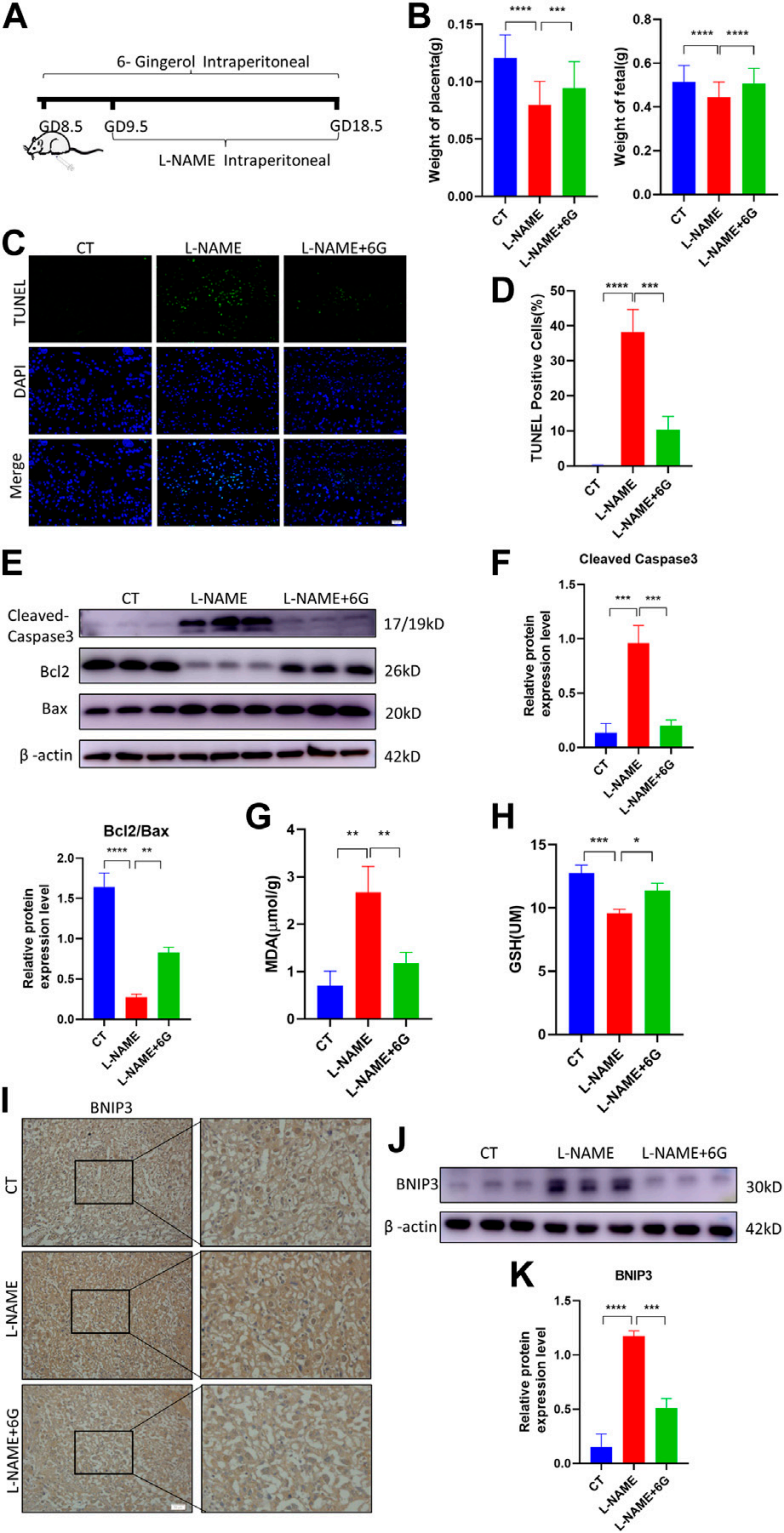
6-Gingerol ameliorated PE-like symptoms *in vivo* and reduced placental injury. **(A)** Schematic depiction of the experimental settings. Pregnant mice were divided into three groups: CT (n = 6), L-NAME (n = 6), and L-NAME + 6G (n = 6). Mice in the L-NAME and L-NAME + 6G groups were administered a subcutaneous injection of 125 mg/kg/day L-NAME from GD 9.5 to GD 18.5, and those of the L-NAME + 6G group were also intraperitoneally administered with 2 mg/kg 6-gingerol from GD 8.5 to GD 18.5. The mice were killed at GD 18.5. **(B)** Placental and fetal weights of dams in each group. **(C, D)** Representative placental images and corresponding semiquantification of the TUNEL assay. Scale bar, 50 μm. **(E, F)** Western blots and corresponding semiquantification of cleaved caspase-3, Bcl2, and Bax in the placentae of each group. **(G)** MDA level and **(H)** GSH level of placentas from each group. **(I)** Representative images of BNIP3 of each group placentas detected by immunohistochemical staining. Scale bar, 50 μm. **(J, K)** Western blots and corresponding semiquantification of BNIP3 in placentas of each group. The data are shown as mean ± SD and analyzed by a one-way ANOVA test, followed by the Tukey–Kramer multiple comparison test based on at least three independent experiments. ns, no significance; *p < 0.05, **p < 0.01, ***p < 0.001, and ****p < 0.0001.

To verify the effect of 6-gingerol on placenta injury of PE *in vivo*, a mouse PE model was established by an intraperitoneal injection of L-NAME, which caused fetal growth restriction (FGR), hypertension, and other symptoms of PE. We measured the fetal and placental weights in each group, finding that the L-NAME injection in pregnant mice dramatically reduced the weight of the fetus and placenta. Compared to the L-NAME-injected group, injection of 6-gingerol increased the average fetal and placental weights (Figure 6B). As shown in Figures 6C, D, the TUNEL assay was used to detect the cell apoptosis of placentae in each group. Compared with the CT group, TUNEL-positive cells increased significantly in the L-NAME group, whereas 6-gingerol repressed the apoptotic index significantly. In support of this, WB results showed that the expression of cell apoptosis markers, such as cleaved caspase-3 and BAX, was upregulated, while the expression of Bcl2 was downregulated by L-NAME, which was reversed by 6-gingerol in the L-NAME + 6-gingerol group (Figures 6E, F). Furthermore, L-NAME generated excess ROS, which resulted in oxidative stress in the placenta. As illustrated in Figures 6G, H, compared with the CT group, L-NAME elevated the levels of oxidative stress biomarkers, including MDA and GSH, in the placenta, whereas 6-gingerol reduced these marker levels notably.

Subsequently, we examined the expression of the mitophagy marker BNIP3, which was a major protein playing a crucial role in damage-induced mitophagy. Immunohistochemistry staining and WB analysis showed that the expression of BNIP3 was upregulated in response to L-NAME, which was reversed by 6-gingerol (Figures 6I–K). All the findings suggested that in the placenta of preeclampsia mice, excess ROS induced oxidative stress and immoderate mitophagy, ultimately leading to trophoblast apoptosis, which was reversed by 6-gingerol.

## 4 Discussion

In this study, we proved the protective effect of 6-gingerol on placenta damage of PE through inhibiting excessive mitophagy. We found that 6-gingerol reduced trophoblast apoptosis and ROS production, increased mitochondrial membrane potential, and downregulated mitophagy by reducing the expression of BNIP3 induced by H/R and its downstream LC3 conversion *in vitro.* In addition, 6-gingerol administration increased the fetal and placental weights, and reduced the apoptosis and oxidative stress injury of the placenta in the PE model. To the best of our knowledge, this is the first study to describe a novel mechanism by which 6-gingerol plays an important role in placental injury of PE via BNIP3-activated mitophagy.

6-Gingerol is a bioactive molecule that has been identified to have wide antioxidative stress and anti-inflammatory effects (Kwiatkowska et al., 1999). It has been shown that 6-gingerol was able to degrade ROS. Studies in animal models demonstrated that 6-gingerol can regulate mitochondrial dysfunction, fatty acid oxidation, lipogenesis, and oxidative stress (Wang et al., 2019; Ganjikunta et al., 2022; Wu et al., 2022). In addition, it decreases the level of MDA and increases the activity of the antioxidant enzyme SOD in a concentration-dependent manner (Zhang et al., 2017). Furthermore, 6-gingerol has been shown to participate in regulating autophagy via increasing Beclin1 expression in endothelial cells (Tsai et al., 2020). Consistent with these findings, in the present study, 6-gingerol significantly attenuated H/R-induced trophoblast apoptosis *in vivo* and *in vitro.*

During the pathogenesis of PE, oxidative stress plays a central role and contributes to the regulation of trophoblast apoptosis. Oxidative stress involves ROS, O_2_^−^, H_2_O_2_, and the hydroxyl radical. It is well known that antioxidant imbalance results in increasing placental production of ROS and reduction of antioxidants, such as SOD, which act as inhibitors of ROS and free radical scavengers in PE placentae (2010). Studies both in human and animal models have suggested that placental and/or fetal hypoxia may be a pathogenetic factor of PE, which is also the cause of the imbalance in pro-oxidant/anti-oxidant activity (Kerley et al., 2018; Liu M. et al., 2021; Guerby et al., 2021). L-NAME has also been used to induce a preeclampsia-like phenotype in mice, including hypertension, proteinuria, fetal growth restriction, and placental impairment (Chen et al., 2019). According to the literature, NO could protect against superoxide-derived ROS and oxidative damage (Wink et al., 1995; Chang et al., 1996; Chen et al., 2019). Therefore, as a NO synthase inhibitor, L-NAME gives rise to oxidative stress injury in the placenta, which has been demonstrated in the present study. In addition, many studies have been carried out on trophoblast cell models to elucidate the origin of oxidative stress, specifically H/R-treated HTR8/SVneo cells (Gu et al., 2022; Zhang and Zhang, 2022). Several studies suggested that placental lipid peroxides increased and placental antioxidant protective effects decreased in PE (Vaka et al., 2021). Consistent with these findings, we demonstrated that production of ROS increased and MDA, LDH, and SOD decreased both in trophoblast exposed to H/R and PE-like mice. The TUNEL and flow cytometry assays indicated that oxidative stress resulted in increased apoptosis in PE placentae, PE trophoblasts, and mouse models. Furthermore, 6-gingerol reduced cytoplasmic and mitochondrial ROS production, involving lower levels of MDA and LDH and a higher level of SOD. In addition, oxidative stress-induced apoptosis could also be attenuated by 6-gingerol. In accordance with these findings, the present study indicated that 6-gingerol restored oxidative stress injury and its apoptotic consequences in the placenta in *in vivo* and *in vitro* models of PE.

Mitochondria are the main source of endogenous ROS with its capacity of oxidative phosphorylation. Mitochondrial electron transport chain and ATP synthesis play a pivotal role in maintaining oxidative homeostasis and modest production of mtROS. However, damaged mitochondria produce excessive ROS as a cytotoxic factory when cells suffer from adverse environmental stresses (Willems et al., 2015). We demonstrated that 6-gingerol played the same role as the specific mitochondrial ROS scavenger MitoTEMPO in ROS production, protecting cells from apoptosis, indicating that mitochondria ROS was the main cause of H/R injury of trophoblasts. Therefore, we believe that the action of 6-gingerol is due to its antioxidant property by removing excess mitochondrial ROS. In the present study, mitochondrial dysfunction caused by H/R treatment manifested by the increase in ROS production and decrease in mitochondrial membrane potential, as well as mitochondrial structural lesions. Our study demonstrated that 6-gingerol prevents H/R-induced trophoblast apoptosis by maintaining the redox balance and defending the oxidative stress.

Mitophagy is defined as the selective autophagic degradation of damaged or surplus mitochondria by autophagosomes and lysosomes in order to maintain the number and function of healthy mitochondria (Pickles et al., 2018). There are two mitophagy regulatory pathways: ubiquitin-dependent and -independent. As the major regulator in the ubiquitin-dependent pathway, PINK1 and Parkin play crucial roles in damage-induced mitophagy (Chen and Dorn, 2013). In addition, BNIP3 serves as a mitophagy receptor in the ubiquitin-independent pathway, interacts directly with LC3 or recruits Parkin, and participates in hypoxia-mediated mitophagy (Fu et al., 2020). Mounting evidence suggests that ubiquitin-independent mitophagy may play a more crucial role in PE compared with the ubiquitin-dependent pathway (Peng et al., 2022). Zhou et al. showed that BNIP3 was downregulated along with autophagic dysfunction and mitochondria injury in PE placentae (Zhou et al., 2021). In contrast, the expression of BNIP3 was significantly higher in PE placentae compared to the term control (Vangrieken et al., 2021). This inconsistency is largely due to the sample selection point in time. In our study, we found that BNIP3 was upregulated in the placenta of premature PE compared with the term control. Additionally, the effects of mitophagy on H/R models established with different cells may yield contradictory results (Peng et al., 2019; Fu et al., 2020; Lee et al., 2020). Our data suggested that the expression of BNIP3 was increased, followed by the increased LC3II/LC3I levels in the trophoblast under H/R treatment and the placenta of PE-like mice, indicating the activation of mitophagy. Interestingly, the levels of P62 did not decrease as LC3 increased, implying the block of autophagy flux. In addition, the colocalization of the GFP-labeled mitochondria and RFP-labeled LC3 illustrates the mitochondrial fusion with autophagosomes, indicating that mitophagy was promoted. The features of mitophagy attributed to H/R treatment in trophoblasts could be attenuated by 6-gingerol or MitoTEMPO, as evidenced by not only the decrease in BNIP3 and LC3II/LC3I but also the reduction of mitophagosomes. Moreover, the same trend of the expression of BNIP3 was shown in the placenta of PE-like mice as those *in vitro.* These data demonstrated that 6-gingerol inhibits excessive mitophagy by regulating the BNIP3-LC3 pathway in PE.

There are some limitations to this study. The maintenance of balance between survival and apoptosis of trophoblasts involves a variety of related cellular interactions and complex regulatory networks. The therapeutic effects of 6-gingerol on placental injury in PE are multifaceted and need to be further explored. In addition, trophoblast-specific silencing of BNIP3 in mice may help confirm the role of 6-gingerol in BNIP3 regulation in the development of PE.

In summary, our current study shows that BNIP3-dependent mitophagy and apoptosis of trophoblasts are activated in human PE placentae. 6-Gingerol prevents H/R-induced apoptosis via suppressing ROS-mediated BNIP3-dependent mitophagy in placental trophoblasts. In conclusion, our findings provide evidence for the application of 6-gingerol as a potential drug for the treatment of PE.

## Data Availability

The original contributions presented in the study are included in the article/Supplementary Material; further inquiries can be directed to the corresponding authors.
