# Supplementary figures and images for: 6-Gingerol alleviates placental injury in preeclampsia by inhibiting oxidative stress via BNIP3/LC3 signaling-mediated trophoblast mitophagy
Source: Front Pharmacol. 2023 Oct 13;14:1243734. doi: 10.3389/fphar.2023.1243734 (PMC10611501; doi:10.3389/fphar.2023.1243734)

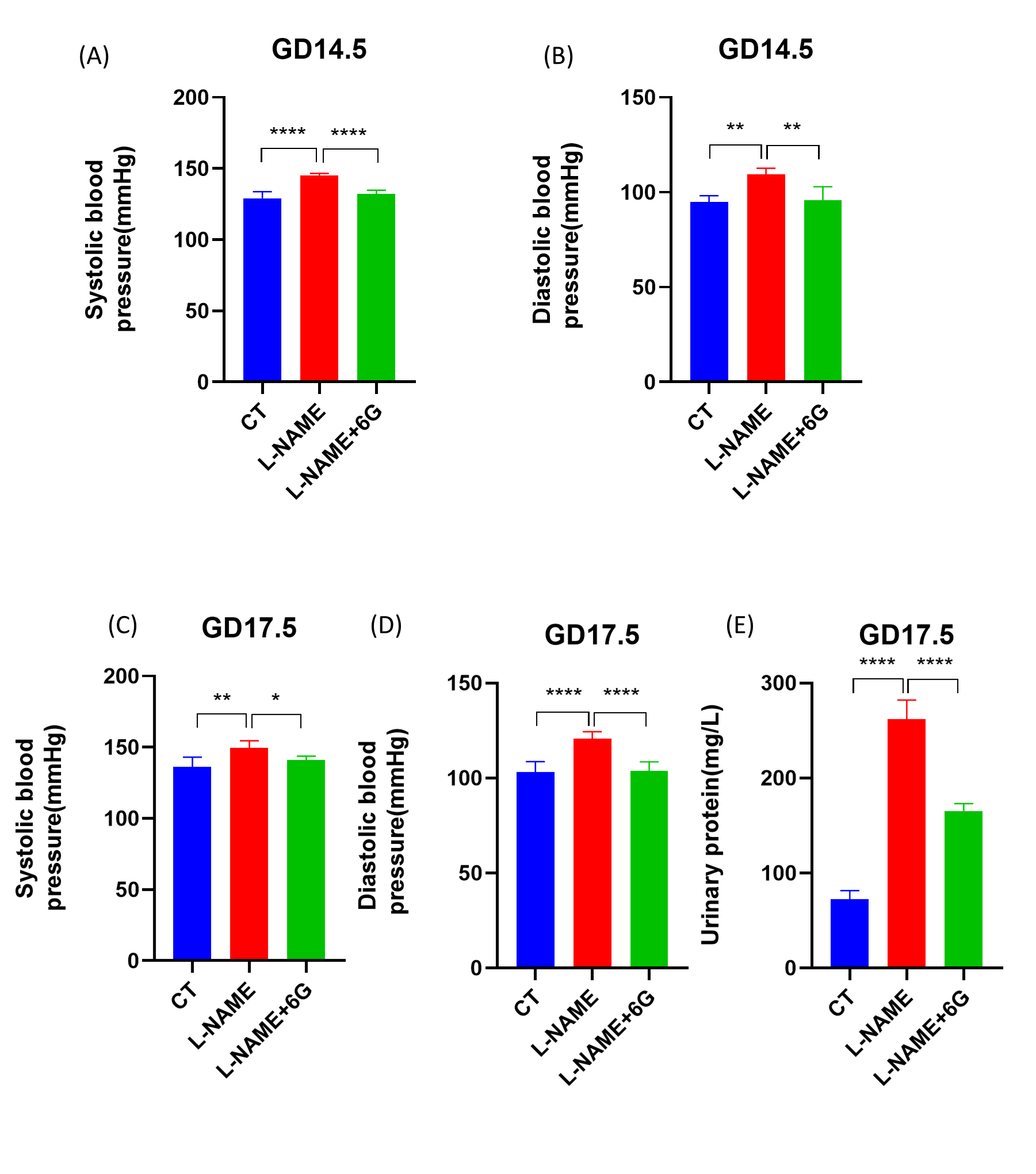

Supplement: Supplementary file 1 [file Image2.TIF]

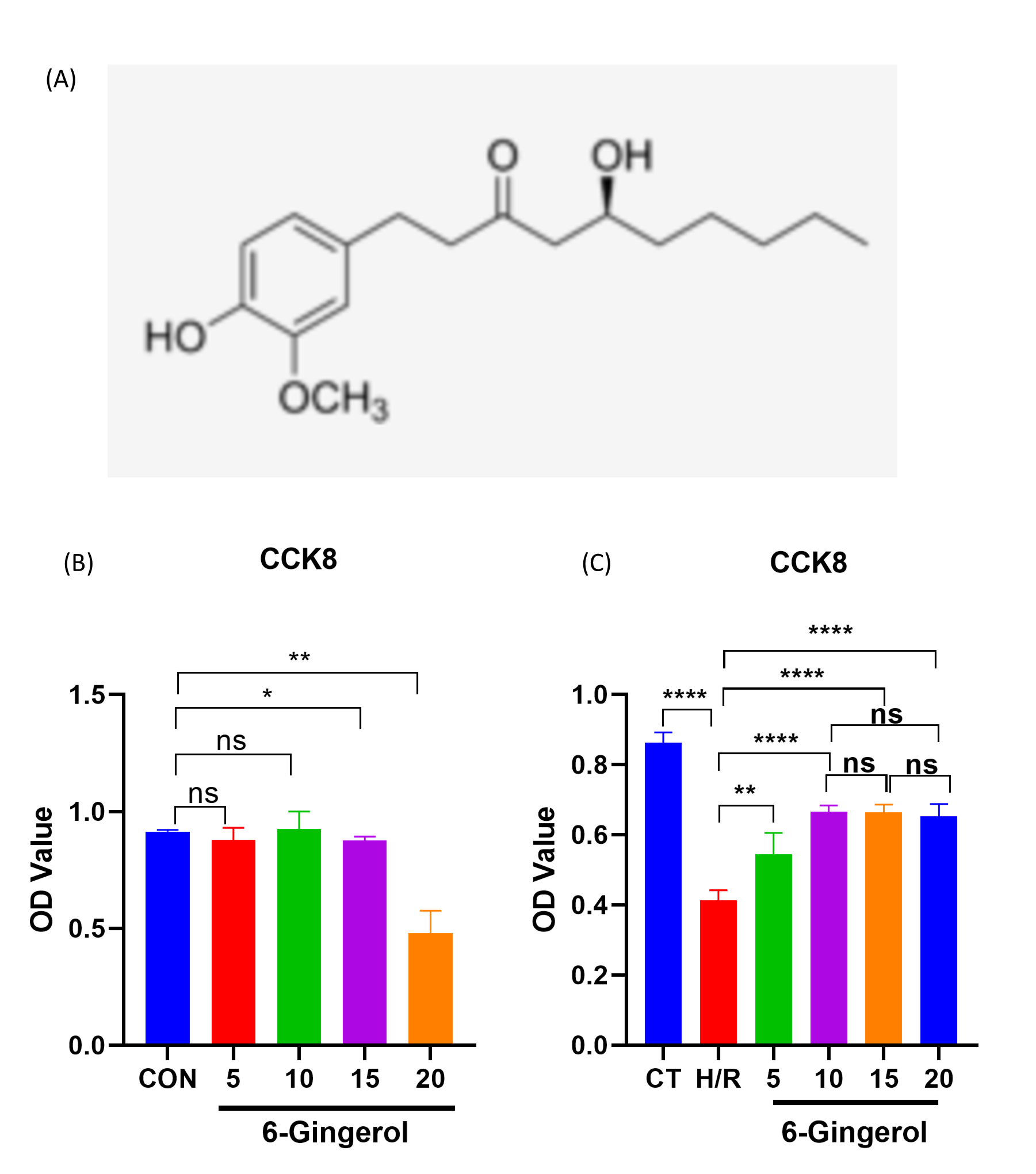

Supplement: Supplementary file 2 [file Image1.TIF]
